# A novel function for vimentin: the potential biomarker for predicting melanoma hematogenous metastasis

**DOI:** 10.1186/1756-9966-29-109

**Published:** 2010-08-11

**Authors:** Man Li, Baogang Zhang, Baocun Sun, Xuan Wang, Xinchao Ban, Tao Sun, Zhiyong Liu, Xiulan Zhao

**Affiliations:** 1 Department of Pathology, The key lab of cancer prevention and treatment, Tianjin Cancer Institute and Hospital, Tianjin Medical University, Tianjin 300060, P.R. China; 2 Department of Pathology, Tianjin Medical University, Tianjin 300070, P.R. China; 3 Department of Digestive, The Second Hospital of Tianjin Medical University, Tianjin 300211, P.R. China; 4 Tianjin Children Hospital, Tianjin 300074, P.R. China

## Abstract

**Background:**

The incidence of malignant melanoma (MM) was occurring at a faster rate than for most neoplasm worldwide, and melanoma metastasis is still the most formidable problem. So it is necessarily to find some biomarkers associated with melanoma metastasis.

**Methods:**

In our study, 8 spontaneous lung metastatic mice models were created by B16F10 subcutaneously transplantation. The differential protein profiles of two kinds of subcutaneous transplanted tumor tissues, which was parental B16F10 (B16 group) and corresponding lung metastases (B16M group) were detected by two-dimensional differential in-gel electrophoresis (2D-DIGE) combined with matrix-assisted laser desorption/ionization time-of-flight mass spectrometry (MALDI-TOF/MS). Western blotting was used to validate the results, and the clinical significance of individual protein was detected furtherly in a set of human samples.

**Result:**

In this study, thirty proteins were found to be differentially expressed (ratio > 2 or < -2, *P *< 0.01) and thirteen of them were identified by MS. Highly expressed proteins in B16M group included cytoskeleton/structure proteins (vimentin, gamma-actin, β-actin, laminin binding protein), the chaperone family of proteins (heavy-chain binding protein, Bip), immunoproteasome assembly (proteasome activator REG alpha) and others involved in glycolysis activity (PGK1, enolase, TPI, human skeletal muscle GAPDH) and protein transport (myoglobin). Vimentin was significantly up-regulated in B16M group compared with B16 group which was validated by western blotting. Immunohistochemistry was performed in a set of clinical samples, the results showed that over-expression of vimentin was frequently observed in primary melanoma patients with hematogenous metastasis (*P *< 0.05), not associated with lymph node metastasis (*P *> 0.05). The presence of TNM stage was a independent indicator of poor prognosis for melanoma patients (*P *= 0.004).

**Conclusion:**

The aberrant immunohistochemical expression of vimentin in primary melanoma tissues may help to call attention for patients with high risk of hematogenous metastasis. That might be as a novel metastatic indicator for melanoma. In a word, vimentin is not only the dignostic marker but also the hematogenous metastasis predictor for melanomas clinically.

## Background

The numbers of malignant melanoma (MM) cases worldwide are increasing faster than any other cancers. It is estimated that the 68,720 new cases of MM will be diagnosed in the United States in 2009 according to SEER Stat Fact Sheets from NCI report [1]. MM is characterized by its intensive metastatsis, therapy-resistant and high mortality. One person dies per hour from metastatic melanoma [2]. Hence tremendous research efforts have been thrown into seeking some biomarkers of metastasis-forecasting for melanoma. Some studies of using high-throughout gene microarray have revealed several putative genes associated with melanoma metastasis, such as SPP-1, MITF, CITED-1, GDF-15, c-Met and so on [3], but none of them was tested the signature in clinical materials.

Recently, novel technology linked with the Human Genome Database, i.e. proteomics has been generally utilized to identify protein biomarkers associated with tumor development and progression. 2D-DIGE (two-dimensional differential in-gel electrophoresis) has higher resolution compared with traditional 2-DE (two-dimensional polyacrylamide gel electrophoresis), which is an advanced quantitative proteomics technology that is of great sensitivity and accuracy [4]. It is a method of prelabeling fluorescent cyanine dyes (Cy2, Cy3, Cy5) to different samples prior to 2-DE. Therefore, different samples can be labeled with the different dyes and separated in the same 2D gel. This technique enables the same internal standard in every gel so as to overcoming the intergel variation. Thus accurate quantitation of differences between samples could be accomplished by 2D-DIGE with high reproducibility and reliability [4].

B16 was derived from a spontaneous melanoma in a C57BL/6J mouse. The subline of B16-F10 was arised from the lung metastasis of the parent B16 line in *vivo *after *i.v*. injection and subsequently cultured in *vitro *after 10 cycles of lung colony formation [5]. Usually, there are two ways to establish lung metastasis, i.e. spontaneous metastasis by inoculation of tumor cells subcutaneously and experimental metastasis by injection of tumor cells directly into the bloodstream. The former one may be better to reflect the metastatic process of the human being than latter. Therefore, using the subcutaneous transplanted tumor tissues from parental B16-F10 (B16 group) and corresponding lung metastases (B16M group) as proteomic objectives may be the most directly and persuasively way to discover metastatic biomarkers for melanoma.

The aim of this study was to investigate novel proteins involving in the metastasis of melanoma by using 2D-DIGE analysis followed by MALDI-TOF/TOF-MS. Furthermore, we examined the properties of these proteins to be metastatic biomarker candidates. The significant protein was successfully validated by immunohistochemistry in 70 primary melanoma cases. This is the first report to confirm the proteomic results in the bulk of clinical specimens.

## Materials and methods

### Cells and animals

Mouse melanoma B16-F10 cells were offered by Tianjin Cancer Hospital. The procedure of engrafted melanoma cells was performed as same as Sun described previously [6]. Till the commence of our study, eight spontaneous metastatic models (B16M group) have been created, and the lungs with metastases have been inoculated into the mice groin to be passaged subsequently. The individual passage times were different from 18 to 33 until the experimental tissues collection.

All six- to eight-week old C57BL/6J mice were purchased from the Animal Center Academy of Military Medical Science. Eight mice were inoculated with B16-F10 suspensions subcutaneously as control group (B16 group). Fifteen days after inoculation, the mice were sacrificed after tumors were harvested. The tumor samples were quickly frozen in liquid nitrogen and kept at -80°C for further analysis.

### Sample preparation and Cy-dye labeling

The frozen tumor samples from two groups were grinded into fine powder in liquid nitrogen and homogenized in lysis buffer (7M urea, 2M thiourea, 4% CHAPS, 10 mM of Tris, 5 mM of magnesium acetate, a complete proteinase inhibitor cocktail tablet per 50 mL lysis buffer), and then solubilized by sonicator (Microson TM Ultrasonic Cell Disruptor, USA) on ice for 1 min. The samples were incubated for 30 min at room temperature with repeated vortexing. They were then centrifuged at 12 000 × g for 40 min at 20°C. The supernatants were saved and total protein concentration was determined with the Bradford assay kit (BioRad). Fifty ug of individual sample lysates were labeled with Cy3 or Cy5 (200 pmol), and equal quantities samples mixed was labeled with Cy2 as the internal pool standard on all gels to aid protein-spot matching cross-gel. Samples were reverse-labeled in order to eliminate either sample-dependent or dye-dependent bias. The labeling process was carried out in the dark on ice for 30 min, and terminated with 1 ul of 10 mM lysine for 10 min on ice. These differently-labeled protein samples were then mixed for 2D-DIGE analysis.

### 2D-DIGE

2D-DIGE was performed as same as Zhang described earlier [7]. Briefly the proteins were applied to IPG strips (pH 3-10, NL, 24 cm) and first-dimension isoelectric focusing (IEF) was performed using an Ettan IPGphor System (GE Healthcare). The strips which were treated with reduction and alkylation steps were overlayered onto 12% SDS polyacrylamide gels (20 × 24 cm) for second dimensional protein separation by using an Ettan DALT Twelve System (GE Healthcare). After SDS-PAGE, the Cy2, Cy3, and Cy5-labeled images were scanned by a laser scanner (Typhoon 9410, GE Healthcare) in fluorescence mode at appropriate excitation/emission wavelengths of 488/520, 532/580, and 633/670 nm respectively.

### Image analysis

The images were analyzed by using DeCyder Differential Analysis Software v6.0 (Amersham GE Healthcare) to detect, quantify and normalize the protein spots intensities in each gel. Differential in-gel analysis (DIA) module was used to detect the merged images of Cy2, Cy3 and Cy5 for each gel, while biological variation analysis (BVA) module was used to automatic match all protein-spot maps. The Cy3/Cy2 and Cy5/Cy2 DIA ratios were used to calculate average abundance changes and paired Student's t-test was conducted. The differential protein spots (ratio > 2 or < -2, *P *< 0.01) which were statistically significant were selected for furthrt identification.

### Spot digestion and MALDI-TOF analysis

Picking the spots, in-gel digestion and MS protein analysis were described as Zhang [7]. Briefly, separate preparative gels which were fixed in 30% v/v methanol, 7.5% v/v acetic acid and stained with colloidal Coomassie Brilliant Blue were used to acquire enough amounts of proteins. Excision of selected protein spots which were interested and confirmed by the 2D-DIGE/DeCyder analysis was subsequently performed with an Ettan Spot Picker. The protein containing gel pieces were discolored with 50% ACN and 25 mM of ammonium bicarbonate, then reduced and alkylated in 10 mM of DTT and 55 mM of iodoacetic acid gradually. The samples were dried by a vacuum centrifuge and were thoroughly incubated with the digestion buffer (linear-gradient Trypsin, a final concentration of 0.01 mg/mL in 25 mM of ammonium bicarbonate) for 16 h at 37°C. After digestion, the samples were centrifuged and the supernatants were removed, vacuum-dried and redissolved in 50% ACN and 0.1% TFA until analysed by MS.

Mixtures of tryptic peptides were eluted onto the 192-well MALDI sample plates with equal amounts of the matrix solution (7 mg/mL CHCA in 0.1% TFA, 50% ACN). Samples were then analyzed by an ABI 4700 Proteomics Analyzer MALDI-TOF/TOF mass spectrometer (Applied Biosystems, USA) to get the peptide mass fingerprint (PMF). Cysteine carbamidomethylation and methionine oxidation were considered as variable modifications. A maximum number of one missed cleavage per peptide was allowed. Precursor error tolerance was set to < 0.1 Da and MS/MS fragment error tolerance < 0.2 Da. When a single spot represented diverse proteins, the proteins composed of highest number of peptides were regarded as corresponding ones. MASCOT search engine (Matrix Science, London, U.K.) was employed to match peptide and search protein against the NCBInr database. Proteins with score value over 60 were positively identified.

### Western blotting

Protein samples were separated by 10% SDS-PAGE gels and then transferred to PVDF membranes. After blocked with 5% defatting milk for 1 h at 37°C, the membranes were incubated with anti-vimentin (1:1000, Thermo Scientific, USA) at 4°C overnight. After washing with 0.5% PBS-T (PBS with Tween-20) for three times, the membranes were incubated with a horseradish peroxidase-conjugated secondary antibody for 1 h at 37°C. Membranes were washed again with PBS-T. The signals were detected by using the Western blotting chemiluminescent kit (Pierce), quantified by densitometry and analyzed by using Quantity One image analysis system (BioRad). The detection of β-actin (1:5000, Santa Cruz, USA) was used as the inner control.

### Patients

Paraffin-embedded melanoma specimens (n = 70) were obtained from the Tianjin Cancer Hospital from 1998 to 2003. Detailed pathological and clinical data were collected and none of the patients had received treatment before operation. Clinical outcome was followed from the date of surgery to the date of death or until Jan, 2009. The summary of the clinicopathological data of the cases is shown in Table 1. Of 70 enrolled cases, 43 males and 27 females (mean age, 54.96 ± 12.60 years). The sites of melanomas were trunk (13/70), limbs (27/70), head and neck (13/70), digestive system (8/70) and genital system (9/70) respectively. We categorized them into two groups: cutaneous melanoma (53/70) and extra-cutaneous melanoma (17/70). The survival durations ranged from 1 to 113 months (mean, 34.90 ± 27.42 months). Primary melanoma with hematogenous metastasis was observed in 29 cases. This study was approved by the ethics committee.

**Table 1:** Correlation of vimentin expression with clinicopathologic features of 70 primary melanoma patients

Patients Characteristics	Factors	n	vimentin expression		χ^2^	*p *value
					
(N = 70)			low	high		
Age(y)	< 60	43	21	22	0.128	0.808
	≥60	27	12	15		
Gender	Male	43	15	12	1.248	0.328
	Female	27	18	25		
Location	Cutaneous	45	22	23	0.154	0.804
	Extra-cutaneous	25	11	14		
TNM Stage	I	16	10	6	2.145	0.342
	II	24	11	13		
	III	30	12	18		
Lymph node metastasis	positive	28	12	16	0.344	0.629
	negative	42	21	21		
Hematogenous metastasis	positive	29	8	21	7.599	0.008*
	negative	41	25	16		

### Immunohistochemical staining of patients samples

Seventy formalin-fixed, paraffin-embedded melanoma patients samples were cut into 4 μm sections and dried overnight at 65°C. The sections were deparaffinized in xylene and rehydrated through graded alcohols into water. Endogenous peroxidase was blocked with 3% hydrogen peroxid for 20 min in the dark chamber. Microwave antigen retrieval was performed using citrate buffer (0.01 M citric acid, pH 6.0) for 20 min at 100°C in a microwave oven. After rinsing with PBS, the slides were incubated with anti-vimentin (1:100, Thermo Scientific, USA) overnight at 4°C. Visualization was performed using the diaminobenzidine (Zhongshan, Beijin, China) according to the manufacturer's instructions. Appropriate positive and negative controls were established. The sections were counterstained with hematoxylin in the end.

The positive expression of vimentin was yellow stain in the cytoplasm of melanoma cells. Ten "hot spots" under high-power fields were selected randomly, and 100 cells per field were counted. The average percentage of positively stained cells of 10 fields was converted into a score as follows: 0 for < 20%, 1 for < 40%, 2 for < 60%, and 3 for > 60%. A score between 2 and 3 was considered to be strong expression.

### Statistic analysis

The statistical analysis was conducted using SPSS version 13.0 (SPSS, Chicago, IL, USA). *P*-value less than 0.05 was defined as significant level. Values are shown as mean ± SD or percentages. The χ^2 ^test, the Student's *t*-test and the Mann-Whitney test were used in our study. Kaplan-Meier survival analysis and log-rank test were performed to compare the survival time between each group. Multivariate survival analysis was performed using the Cox proportional hazards model.

## Results

### 2D-DIGE Images of Proteins

**Figure 1:**
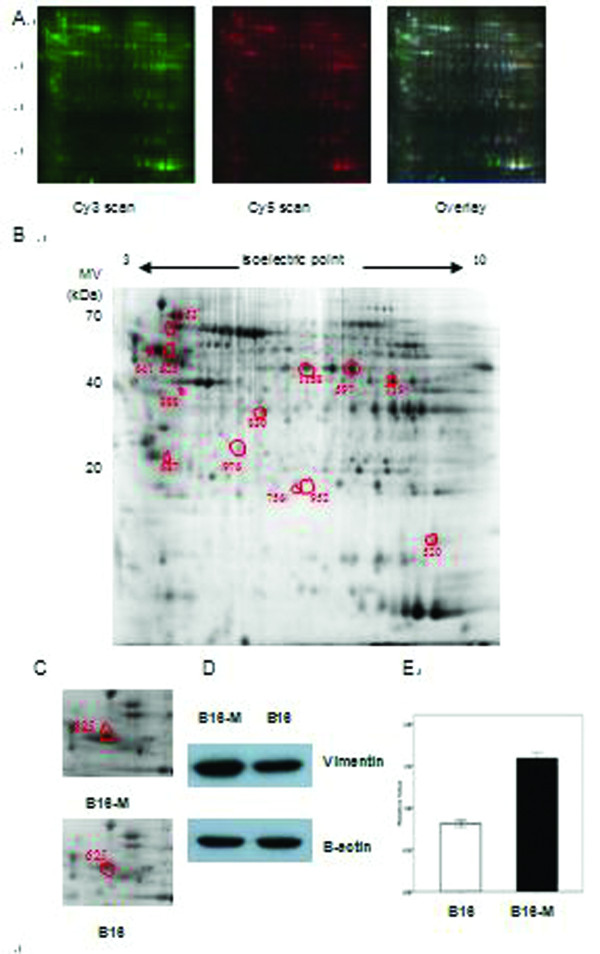
**The images of representive 2D-DIGE  and validation of vimentin**. (A) A representative 2D-DIGE gel images. The extracted proteins were labeled with fluorescent dyes and separated by 2D-DIGE. B16M group was labled with cy3, B16 group was labled with cy5. (B) A representative two-dimensional gel image. Differential expressed proteins that have been successfully identified by MALDI-TOF/MS (p ≤ 0.05, protein fold≥2) are circled and numbered. The spot numbers correspond to those proteins listed in Table 1. (C) The magnified protein spot images of vimentin in 2D gel showing the significant over-expression in B16M group compared with B16 group. (D) Western blotting shows changes in expression levels of vimentin in B16M group and B16M group; *β*-actin is used as the internal loading control. (E) Histogram showing the relative expression levels of vimentin in eight pairs of B16M and B16 tissues, as determined by densitometric analysis (*p *= 0.021).

Protein profiles which were potentially involved in metastasis were analyzed by 2D-DIGE which was repeated independently for three times under identical condition. The threshold of proteins differential expression was set at great than 2-fold, and the P value of < 0.01 of *t*-test was regarded as statistical significance. Thirty spots across all images were differential significantly. They were subsequently excised, subjected to trypsin digestion in-gel and analyzed by MALDI-TOF/TOF- MS. Thirteen proteins of them were successfully identified by PMF analysis and peptide sequences analysis in the NCBInr database (Table 2). Of the 13 protein spots, 11 were higher abundance and 2 were at lower levels in the metastatic group. Highly expressed proteins in B16M group included cytoskeleton/structure proteins (vimentin, gamma-actin, β-actin, laminin binding protein), the chaperone family of proteins (heavy-chain binding protein, Bip), immunoproteasome assembly (proteasome activator REG alpha) and others involved in glycolysis activity (PGK1, enolase, TPI, human skeletal muscle GAPDH) and protein transport (myoglobin). MALDI-TOF/TOF-MS analysis and database matching identified spot 625 was vimentin with high sequence coverage and mass accuracy (Figure 1A-C).

**Table 2:** The list of differential proteins identified by MS

spot no.	Acession number	identified protein	average ratio	M.W(Da)	PI	Protein coverage	Protein score C.I.%	Functional classification
520	gi|17389985	MB protein [Homo sapiens] myoglobin	2.13	10863	9.24	40%	100	Transport
597	gi|31873302	hypothetical protein [Homo sapiens]	-3.24	47063	7.57	8%	99.993	
625	gi|47115317	VIM [Homo sapiens]	2.06	53547	5.09	27%	97.337	Cytoskeleton
641	gi|16552261	unnamed protein product [Homo sapiens]	-2.7	47459	5.01	42%	100	
687	gi|178045	gamma-actin[Homo sapiens]	2.26	25862	5.65	14%	100	Cytoskeleton
719	gi|48145549	PGK1 [Homo sapiens]	3.08	44574	8.30	28%	100	Glycolytic Enzymes
756	gi|1125065	laminin-binding protein laminin receptor	3.05	14104	7.03	16%	98.157	Cytoskeletal/structural protein
830	gi|230867	Chain R, Twinning In Crystals Of Human Skeletal Muscle GAPDH	4.16	35853	6.60	11%	100	Glycolytic Enzymes
888	gi|15277503	ACTB protein [Homo sapiens] b-actin	3.09	40194	5.55	17%	100	Cytoskeleton
952	gi|2780871	Chain B, Proteasome Activator Reg(Alpha)	3.71	16285	7.14	14%	99.989	Immunoproteasome assembly
976	gi|999892	Chain A, Crystal Structure Of Recombinant Human Triosephosphate Isomerase	4.12	26522	6.51	22%	99.594	Glycolytic Enzymes
1153	gi|6470150	BiP protein [Homo sapiens]	3.12	70888	5.23	41%	100	the chaperone family of protein
1158	gi|4503571	enolase 1 [Homo sapiens]	4.72	47139	7.01	41%	100	Glycolytic Enzymes

* average ratio, B16M group/B16 group

### Validation of vimentin expression by western blotting

Western blotting was performed to verify the differential expression of vimentin in eight pairs of B16M group and B16 group. Equal expression of β-actin as internal standard was to identify the same protein loading. As shown in Figure 1D-E, vimentin was significantly up-regulated in B16M group compared to B16 group (P < 0.05), which was consistent with the 2D-DIGE results.

### Expression of vimentin in melanoma patients

**Figure 2:**
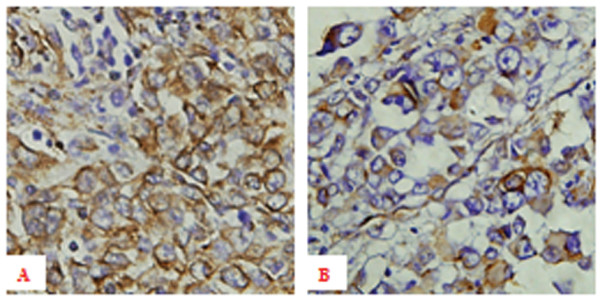
**The immunohistochemical expression of vimentin in melanoma patients**. (A) High expression of vimentin in primary melanoma tissue with hematogenous metastasis. ×400 (B) Low expression of vimentin in primary melanoma tissue without hematogenous metastasis. ×400.

We further detected the expression of vimentin using immunohistochemistry in 70 primary malignant melanoma patients to evaluate its clinicopathological significance. The differential expression of vimentin was shown in Figure 2A-B. Primary melanomas with overexpression of vimentin tends to have a more hematogenous metastasis incidence (*P *< 0.05). There is no statistical significance between overexpression of vimentin with age, gender, tumor location, TNM stage and lymph node metastasis (Table 1). Cox proportional hazards model analysis was performed and showed that the presence of TNM stage was a independent indicator of poor prognosis for melanoma patients (*P *= 0.004).

## Discussion

Melanoma metastasis is the most insidious and life-threatening. To identify the metastasis-associated biomarkers may help to provide risk assessments and personal therapeutic strategies for melanoma patients. The earlier detection such accurate biomarkers in the primary tumors, the better prognosis and interventional treatments would patients have. Along with the advanced technologies, a series of high-throughput DNA microarray platforms have been applied to identify genic targets associated with metastatic biological phenotypes of melanomas [8-10]. However, the proteome is the functional translation of the genome and can regulate cancer cells behavior directly. Neither the DNA sequences nor the amount of RNA could predict post-translational aberrations resulting from phosphorylation, glycosylation or proteolysis[11]. So it is reasonable that the proteomics should reflect the tumor characteristic more directly than genomics.

Till now, there have been a number of researches focusing on detecting the metastatic biomarkers for melanoma by using the proteomics methodologies [12-14]. The cell lines of different biological features were used as the compared objectives customarily and 2-DE combined with MS were most favorable methods for proteomics. The traditional 2-DE is short of reproducibility owing to gel-to-gel variation. That has been resolved by advanced technique of 2D-DIGE which is of higher sensitivity and reproducibility. In 2D-DIGE, the protein extracts are labeled with fluorescent cyanine dyes, mixed and separated in the same 2D gel where has a unified internal standard [4,15]. For its ascendancy, we applied it instead of the classical 2-DE in this study.

In order to discover metastasis-associated biomarks for melanoma, the research objectives originating from the primary tumors with those corresponding metastases of the same patients are the optimum. Unfortunately, it is too difficult to acquire such specimens clinically. For this reason, we created the mice models bearing spontaneous lung metastasis by using B16-F10 subcutaneously inoculation. That metastatic process could mimic the procedure in the human body. The metastatic "black spots" on the mouse lung were picked out, transplanted into the mouse groin and then growed into transplanted tumor which were passaged sequentially and stably. We compared the differential protein profiles to identify which proteins were varied during the metastatic process.

In this study, thirty proteins were differential expressed statistically between two groups and thirteen of them were successfully identified by MS. Functional analysis demonstrated that proteins of higher abundance in metastatic process were more associated with cytoskeletal structure, glycometabolism, protein folding, and immune response, suggesting that these proteins should be involved in melanoma progression. Several up-regulated proteins, such as laminin binding protein and GRP78 (Bip) have been reported that played important roles in either melanoma progression or various cancers metastasis [16-18]. Furthermore, another individual up-regulated proteins in our study have already been identified as metastatic markers in other types of cancer by using proteomics methods, these were PA28 (proteasome activator alpha) implicated in ovary cancer [19], α-enolase in hepatocellular carcinoma [20], triosephosphate isomerase in lung squamous carcinoma [21] and PGK1 in gastric cancer [22].

The most valuable significance of our study is to discover that vimentin might be served as a potential biomarker for predicting the melanoma hematogenous metastasis by using one set of clinical samples. Vimentin was up-regulated 2.06 folds in the B16M group compared with the B16 group in 2D-DIGE and the result was confirmed by western blotting subsequently. The clinicopathological analysis was performed to detect whether there had differential expression of vimentin in primary tumors with or without hematogenous metastasis by immunohistochemical staining. The data showed that high expression of vimentin was significantly associated with melanoma hematogenous metastasis. There was more occurrence of over expression of vimentin in primary melanomas with hematogenous metastasis (21/29, 72. 41%) compared to non-hematogenous metastasis (16/41, 39.02%). However, the expression of vimentin is not differential significantly between primary melanomas with lymph nodes metastasis (16/28, 57.14%) with non-lymph nodes metastasis (21/42, 50%). So we presume that vimentin should have special biological features in melanoma hematogenous metastasis, not involving in lymph node metastasis. Although cutaneous melanoma is the majority type, extra-cutaneous melanoma is still occupying a small part. Sixteen of the former (16/45) and thirteen of the latter (13/25) were positively for hematogenous metastasis. It seemed that extra-cutaneous melanoma have more occurrence of hematogenous metastasis. The prognostic factors for cutaneous melanoma include Breslow tumor thickness, Clark's level, ulceration and lymph node metastasis [23]. In our study, for cutaneous melanoma and extra-cutaneous melanoma, the TNM stage is an independent indicator of poor prognosis.

Generally, vimentin is usually used as a marker to diagnose human melanoma clinically. But with the increasing knowledge about it, we have known that the extensive function of vimentin are far more than these. Numerous studies relating to proteomics have shown that vimentin was metastasis-associated factor in multiple malignancies, such as prostate cancer [24], breast cancer [25], gastric cancer [26], and galbladder cancer [27]. That suggests that vimentin should play an important role in tumor progression and serve as a potential biomarker for the metastasis. There still have some studies which were concerning of aberrant overexpression of vimentin and its relationship with melanoma metastasis [28,29]. On the whole, we first demonstrated the significant upregulation of vimentin in metastatic melanoma compared to primary cases by proteomics and carried out the clinical verification to evalute whether vimentin is a potential biomarker for predicting the metastasis in melanoma patients.

Vimentin is one of the most familiar members of intermediate filaments (IFs) which is the characteristic of mesenchymal cells. IFs, actin microfilaments and microtubules are three major structural components of the cytoskeleton which are in charge of contraction and migration of cells. In addition, the stucture where vimentin, actin associate with integrins and where vinculin and plectin recruited were termed as the vimentin associated matrix adhesions (VAMs) [30]. Of our results, laminin receptor and actin (β,γ) were all up-regulation in the metastatic group. It revealed that cytoskeleton proteins might be associated with melanoma metastasis intensively. Metastasis is a complicated process, of them adhesion is a prerequisite step by which tumor cells could be easy to migrate, invade and detach from the primary tumour. Recent studies have revealed that vimentin has key roles in adhesion by regulating integrin functions [31]. So it could be as a therapeutic target for melanoma in the future. In addition to this, Vimentin is still the predominant mesenchymal marker which is atypical expressed in the epithelial-mesenchymal transition (EMT). EMT is the process that the epithelial cells acquire the mesenchymal phenotype with more migratory and invasive properties. Resently, more and more attentions have been focused on the EMT which seems to act as a switch for the initial cancer metastasis[32]. Generally, EMT is defined as the upregulation of mesenchymal markers and downregulation of epithelial markers. Till now, there have been some reports to identify that melanoma metastasis were associated with EMT [33,34]. Alonso et al [34] confirmed that the expression of a set of proteins included in the EMT group (N-cadherin, osteopontin, and SPARC/osteonectin) were significantly associated with metastatic development of melanomas using cDNA microarrays. In our MS results, only vimentin and actin were identified up-regulated, no other epithelial markers were identified, that is one shortcoming of our study. So it is merely a hypothesis that vimentin involving in the melanoma metastasis is by EMT progression.

## Conclusions

This is the first report to validate the proteomics results in a set of melanoma samples. Our results showed that increased expression of vimentin might be as a novel metastatic indicator for melanoma. In other words, vimentin is not only the dignostic marker but also the hematogenous metastasis predictor for melanomas clinically. The aberrant immunohistochemistry expression of vimentin in primary melanoma tissues may help to call attention for patients with high risk of hematogenous metastasis. Accordingly, we could have possibility to predict the clinical outcome, and then to provide individual treatment strategies for melanoma patients.

## Abbreviations List

BVA: biological variation analysis; DIA: differential in-gel analysis; EMT: epithelial-mesenchymal transition; IEF: isoelectric focusing; IFs: intermediate filaments; MM: malignant melanoma; PMF: peptide mass fingerprint; 2-DE: two-dimensional polyacrylamide gel electrophoresis; 2D-DIGE: two-dimensional difference gel electrophoresis; vimentin VAMs: associated matrix adhesions

## Competing interests

The authors declare that they have no competing interests.

## Authors' contributions

BGZ, ML and XW carried out experimental procedures and drafted manuscript. TS, XCB and ZYL participated in its design and carried out the molecular experiments. XLZ revised it critically. BCS guaranted the whole study. All authors read and approved the final manuscript.
